# Corticosteroids in the Treatment of Community-Acquired Pneumonia in Adults: A Meta-Analysis

**DOI:** 10.1371/journal.pone.0047926

**Published:** 2012-10-24

**Authors:** Wei Nie, Yi Zhang, Jinwei Cheng, Qingyu Xiu

**Affiliations:** 1 Department of Respiratory Disease, Shanghai Changzheng Hospital, Second Military Medical University, Shanghai, China; 2 Department of Gastroenterology, The 452nd Military Hospital of China, Chengdu, Sichuan, China; 3 Department of Ophthalmology, Shanghai Changzheng Hospital, Second Military Medical University, Shanghai, China; Barcelona University Hospital, Spain

## Abstract

**Background:**

The benefit of corticosteroids in community-acquired pneumonia (CAP) remains controversial. We did a meta-analysis to include all the randomized controlled trials (RCTs) which used corticosteroids as adjunctive therapy, to examine the benefits and risks of corticosteroids in the treatment of CAP in adults.

**Methods:**

Databases including Pubmed, EMBASE, the Cochrane controlled trials register, and Google Scholar were searched to find relevant trials. Randomized and quasi-randomized trials of corticosteroids treatment in adult patients with CAP were included. Effects on primary outcome (mortality) and secondary outcomes (adverse events) were accessed in this meta-analysis.

**Results:**

Nine trials involving 1001 patients were included. Use of corticosteroids did not significantly reduce mortality (Peto odds ratio [OR] 0.62, 95% confidence interval [CI] 0.37–1.04; *P = *0.07). In the subgroup analysis by the severity, a survival benefit was found among severe CAP patients (Peto OR 0.26, 95% CI 0.11–0.64; *P = *0.003). In subgroup analysis by duration of corticosteroids treatment, significant reduced mortality was found among patients with prolonged corticosteroids treatment (Peto OR 0.51, 95% CI 0.26–0.97; *P = *0.04; *I* ^2^ = 37%). Corticosteroids increased the risk of hyperglycemia (Peto OR 2.64, 95% CI 1.68–4.15; *P*<0.0001), but without increasing the risk of gastroduodenal bleeding (Peto OR 1.67, 95% CI 0.41–6.80; *P = *0.47) and superinfection (Peto OR 1.36, 95% CI 0.65–2.84; *P = *0.41).

**Conclusion:**

Results from this meta-analysis did not suggest a benefit for corticosteroids treatment in patients with CAP. However, the use of corticosteroids was associated with improved mortality in severe CAP. In addition, prolonged corticosteroids therapy suggested a beneficial effect on mortality. These results should be confirmed by future adequately powered randomized trials.

## Introduction

Community-acquired pneumonia (CAP) is a common and serious infectious disease associated with high morbidity and mortality. It is the sixth leading cause of death and the most common infectious cause of death worldwide [1]. Despite effective antibiotic therapy, about 12–36% patients admitted to the intensive care unit (ICU) with severe CAP die within a short time [2]. Therefore, the development of an efficacious treatment has important implications for reducing the high mortality.

During infectious pneumonia, inflammatory cytokines, such as interleukin (IL)-6, IL-8 and IL-10 acted as acute phase proteins [3]. A recent study showed that the excess of IL-6 and IL-10 was associated with a high mortality rate in CAP [4]. Corticosteroids are the most effective and widely used anti-inflammatory drugs. An early study demonstrated the association of glucocorticoids with antibiotics attenuated local inflammatory response and decreased bacterial burden in the experimental model of severe pneumonia [5]. In a mouse pneumonia model, Li et al. [6] found that hydrocortisone decreased inflammatory response significantly. In addition, Salluh et al. [7] reported that relative adrenal insufficiency occurred in most of the patients with severe CAP, suggesting underlying benefits of corticosteroids treatment in these patients. Taken together, these facts indicated a potential beneficial effect of corticosteroids in pneumonia.

Recently, a multicenter randomized controlled trial (RCT) performed by Confalonieri et al. [8] demonstrated that hydrocortisone treatment in severe CAP was associated with a significant reduction in mortality. A retrospective study conducted by Garcia-Vidal et al. [9] found that mortality decreased in the patients who received systemic steroids along with antibiotic treatment for severe CAP. Moreover, results from a systematic review showed that administration of corticosteroids in patients with CAP was associated with a lower mortality [10]. However, these findings were not confirmed in the subsequent larger RCTs [11]–[14]. Another recent retrospective study also showed that adjunctive therapy with corticosteroids did not influence the mortality rate [15]. Furthermore, results from a meta-analysis found that participants receiving corticosteroids displayed no significant differences in mortality compared with placebo [16]. Consequently, the benefits of corticosteroids treatment in CAP are still uncertain.

The aim of this meta-analysis was to evaluate the efficacy and safety of corticosteroids adjunctive therapy in the treatment of CAP in adults.

## Materials and Methods

This meta-analysis was performed according to a predetermined protocol described in the following paragraph, using standard systematic review techniques, as outlined by the Cochrane Handbook for Systematic Reviews of Interventions and PRISMA Statement [17], [18].

### Eligibility Criteria and Outcome Measures

Studies fulfilling the following selection criteria were included in this meta-analysis: (1) study design: RCTs, including quasi-RCTs; (2) population: adult patients with CAP; (3) intervention: corticosteroids adjunctive therapy in CAP; (4) comparison intervention: placebo or standard treatment; and (5) outcome variables: mortality. Studies were excluded if: studies enrolled pediatric patients or nosocomial pneumonia patients.

The primary outcome measure was mortality. Secondary outcome measures included: hyperglycemia, gastroduodenal bleeding, and superinfection.

### Information Sources and Search

A Pubmed, EMBASE and the Cochrane controlled trials register search identified RCTs of corticosteroids adjunctive therapy versus control participants published up to May 2012 in any language literature. The electronic search strategy included the terms “steroids”, “glucocorticoids”, “corticosteroids”, “hydrocortisone”, “prednisone”, “methylprednisolone”, “dexamethasone” and “community-acquired pneumonia”. We also searched the reference lists of original reports and systematic review of studies involving CAP to identify studies not yet included in the computerized databases. In addition, we reviewed the cited lists of eligible trials by Google Scholar to ensure that all appropriate studies were included.

### Study Selection

**Figure 1:**
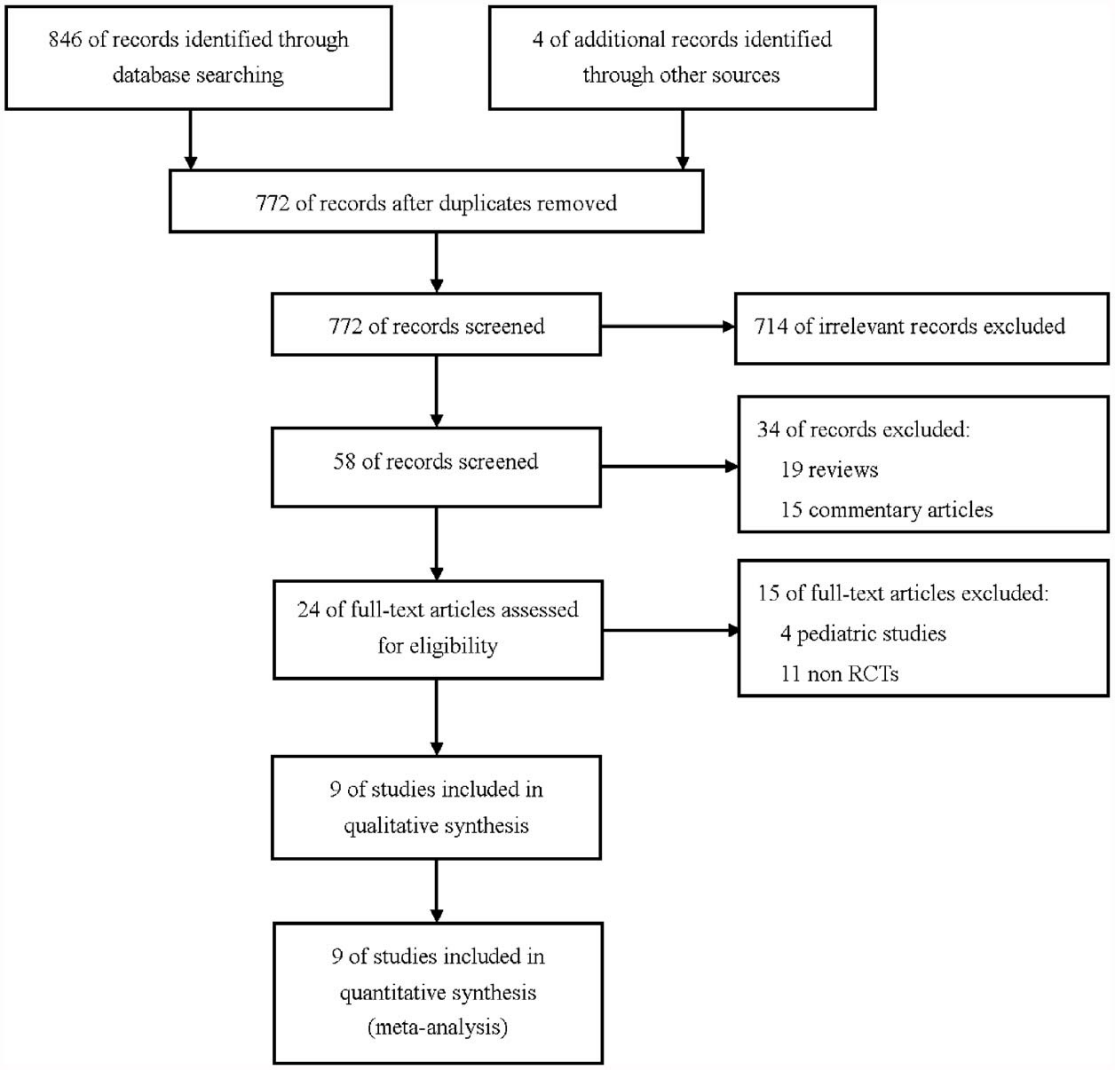
Flow of study identification, inclusion, and exclusion.

A total of 772 studies were retrieved, and the process of identifying relevant trials is shown in **Figure 1**. Among the 772 initially potentially relevant studies, one study was searched from Google Scholar through reviewing the cited lists of eligible trials [14], and three studies were identified from reviews by hand searched [19]–[21]. 748 studies were excluded because of irrelevant, review or commentary articles. After full-text articles assessed for eligibility, fifteen studies were excluded because four studies enrolled pediatric patients and the other eleven studies were non-RCTs. Subsequently, nine RCTs were included in qualitative synthesis and quantitative synthesis.

### Data Collection Process

Two authors independently reviewed full manuscripts of eligible trials, and the relevant data were extracted into predesigned data collection forms. We verified accuracy of data by comparing collection forms from each reviewer. Any discrepancy was resolved by discussion or a third author would assess these articles. The following data were collected from each study: first author, year of publication, study design, location, sample size, participant demographics, corticosteroids doses, duration of corticosteroids treatment and outcome variables. Authors of the included studies were contacted via E-mail if further study details were needed.

### Qualitative Assessment

Methodological quality assessment was independently performed by two of the authors, and any disagreement was resolved by consensus. Risk of bias was evaluated as high, low, or unclear using the Cochrane Risk of Bias Tool for RCTs [17].

### Statistical Analysis

The outcome measure was assessed in an intention-to-treat (ITT) manner. Peto method works well when intervention effects are small, events are not particularly common and the study has similar numbers in two groups [22]. Therefore, mortality and adverse events were analyzed using the Peto method to calculate odds ratio (OR) and 95% confidence interval (CI). Heterogeneity was assessed using the *I* ^2^ test statistic and classified as low (≤25%), moderate (25–50%), and high (>50%). Subgroup analysis was performed by the severity of CAP and the duration of corticosteroids treatment. We defined the prolonged course as corticosteroids treatment for more than 5 days [23]. Prespecified sensitivity analyses were conducted to determine the influence of statistical models (the fixed-effects model and the random-effects model) on effect size. Graphic exploration with funnel plot was used to evaluate the publication bias visually. The Egger’s test [24] was used to assess publication bias statistically. A *P* value <0.05 was considered statistically significant. All statistical tests were performed by using the RevMan 5.1 software (Nordic Cochrane Center, Copenhagen, Denmark) and STATA 11.0 software (Stata Corporation, College Station, TX).

## Results

### Trial Characteristics

Nine clinical trials including 1001 adult patients qualified for inclusion [8], [11]–[14], [19], [20], [25], [26]. 468 patients were allocated to the corticosteroids groups, and 533 patients were allocated to the control groups. Among these trials, five were conducted in Europe, two in North America, one in Africa, and one in Asia. The mean age of the patients ranged from 34 to 72 years. Four trials specifically included severe CAP patients [8], [11], [14], [25]. The remaining five trials included those with mild to severe CAP patients [12], [13], [19], [20], [26]. Four trials were multicenter RCTs [8], [19], [12], [14]. The selected trials used various corticosteroids, including hydrocortisone, prednisolone, dexamethasone, and methyl-prednisolone. The durations of corticosteroids treatment ranged from 1 to 9 days. The characteristics of the trials are shown in **Table 1**.

**Table 1:** Characteristics of included trials.

Author/Year	Study Design	Location	No. Patients	Mean Age (y)	Patient Selection	Corticosteroids Used
Wagner [19]/1956	Quasi-RCT	USA	113	N/A	Mild to severe	Hydrocortisone, 560 mg, 5 d
		Multicenter				
McHardy [20]/1972	Open-label RCT	UK	126	60	Mild to severe	Prednisolone, 20 mg/d, 7 d
		Single center				
Marik [25]/1993	DB RCT	USA	30	34	Severe	Hydrocortisone, 10 mg/kg, 1 d
		Single center				
Confalonieri [8]/2005	DB RCT	Italy	48	64	Severe	Hydrocortisone, 240 mg/d, 7 d
		Multicenter				
Mikami [26]/2007	Open-label RCT	Japan	31	72	Mild to severe	Prednisolone, 40 mg/d, 3 d
		Single center				
Snijders [13]/2010	DB RCT	Netherlands	213	63	Mild to severe	Prednisolone, 40 mg/d, 7 d
		Single center				
Meijvis [12]/2011	DB RCT	Netherlands	304	63	Mild to severe	Dexamethasone, 5 mg/d, 4d
		Multicenter				
Sabry [14]/2011	DB RCT	Egypt	80	62	Severe	Hydrocortisone, 300 mg/d, 7d
		Multicenter				
Fernández-Serrano [11]/2011	DB RCT	Spain	56	63	Severe	Methyl-prednisolone, 620 mg, 9d
		Single center				

DB, Double-Blinded; RCT, randomized controlled trial.

### Quality Assessment

We assigned an unclear risk of bias to one study [19] due to insufficient information regarding randomization and allocation. Despite two studies were open-label RCTs [20], [26], we assigned a low risk of bias to them, as the lack of blinding would be unlikely to affect mortality. Double-blinded RCTs were assigned to a low risk of bias. **Table 2** summarizes the risk of bias.

**Table 2:** Risk of bias summary of included studies.

Author	Randomizationmethod	Allocationconcealment	Blinding of participantsand personnel	Blinding ofoutcome assessment	Incompleteoutcome data	Selectivereporting	Other bias
Wagner [19]	Unclear risk	Unclear risk	Low risk	Low risk	Low risk	Unclear risk	Unclear risk
McHardy [20]	Low risk	Low risk	Low risk	Low risk	Low risk	Unclear risk	Unclear risk
Marik [25]	Low risk	Low risk	Low risk	Low risk	Low risk	Low risk	Unclear risk
Confalonieri [8]	Low risk	Low risk	Low risk	Low risk	Low risk	Low risk	Unclear risk
Mikami [26]	Low risk	Low risk	Low risk	Low risk	Low risk	Unclear risk	Unclear risk
Snijders [13]	Low risk	Low risk	Low risk	Low risk	Low risk	Low risk	Low risk
Meijvis [12]	Low risk	Low risk	Low risk	Low risk	Low risk	Low risk	Low risk
Sabry [14]	Low risk	Low risk	Low risk	Low risk	Low risk	Low risk	Unclear risk
Fernández-Serrano [11]	Low risk	Low risk	Low risk	Low risk	Low risk	Low risk	Low risk

### Primary Outcome

**Figure 2:**
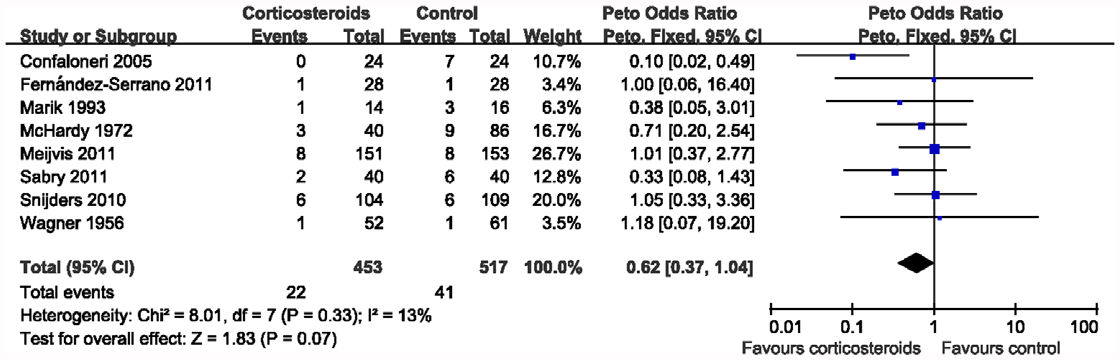
Meta-analysis for the association between mortality and corticosteroids.

Data on mortality was available from eight trials (n = 970). Mortality was not significantly reduced by the use of corticosteroids (Peto OR 0.62, 95% CI 0.37–1.04; *P = *0.07) (**Figure 2**). There was low heterogeneity (*I* ^2^ = 13%).

### Secondary Outcomes

Four trials (n = 488) reported gastroduodenal bleeding events [8], [11], [12], [14]. No significant difference was detected (Peto OR 1.67, 95% CI 0.41–6.80; *P = *0.47; *I* ^2^ = 0%). Three trials (n = 565) reported superinfection events [8], [12], [13]. There was also no difference (Peto OR 1.36, 95% CI 0.65–2.84; *P = *0.41; *I* ^2^ = 71%). Data on hyperglycemia events was available for three trials (n = 573) [11]–[13]. Corticosteroids was associated with more hyperglycemia events (Peto OR 2.64, 95% CI 1.68–4.15; *P*<0.0001; *I* ^2^ = 0%).

### Subgroup Analysis

**Figure 3:**
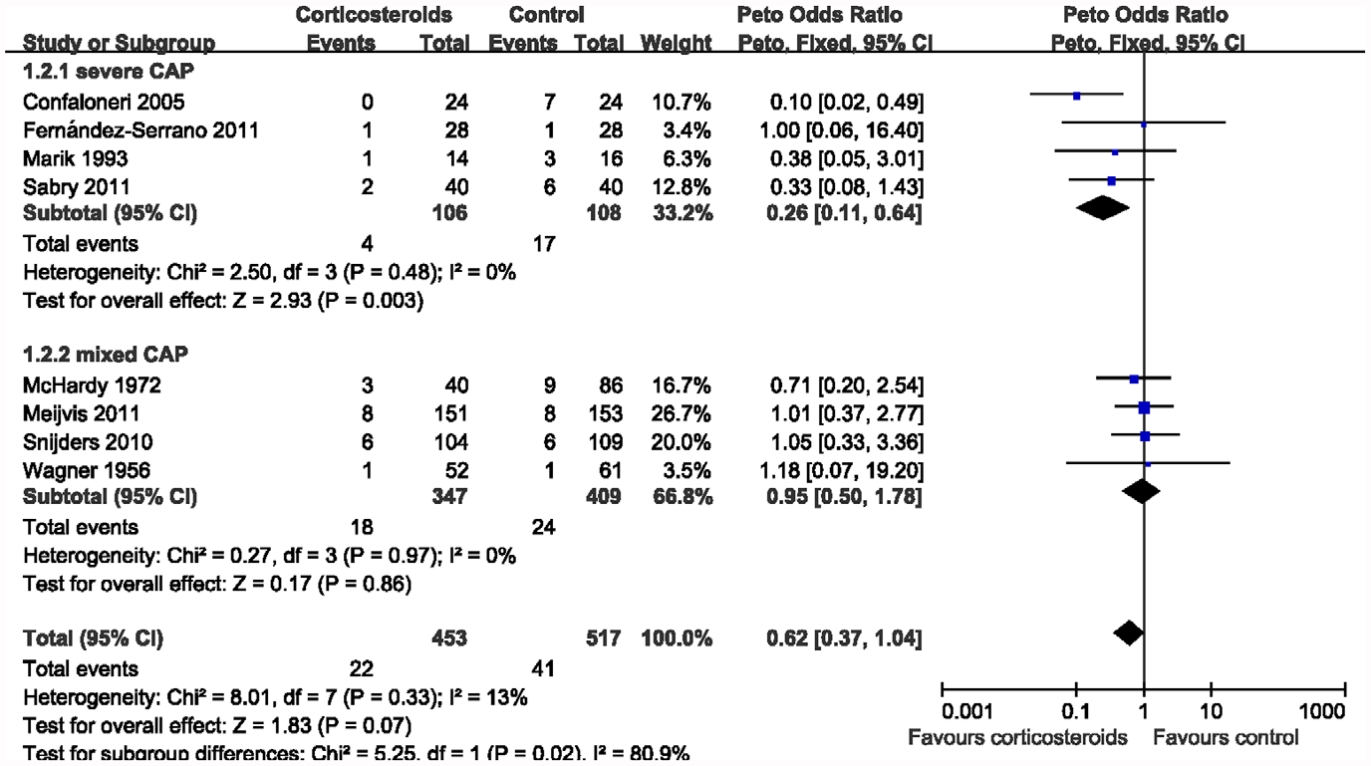
Subgroup analysis according to the severity of CAP.

**Figure 4:**
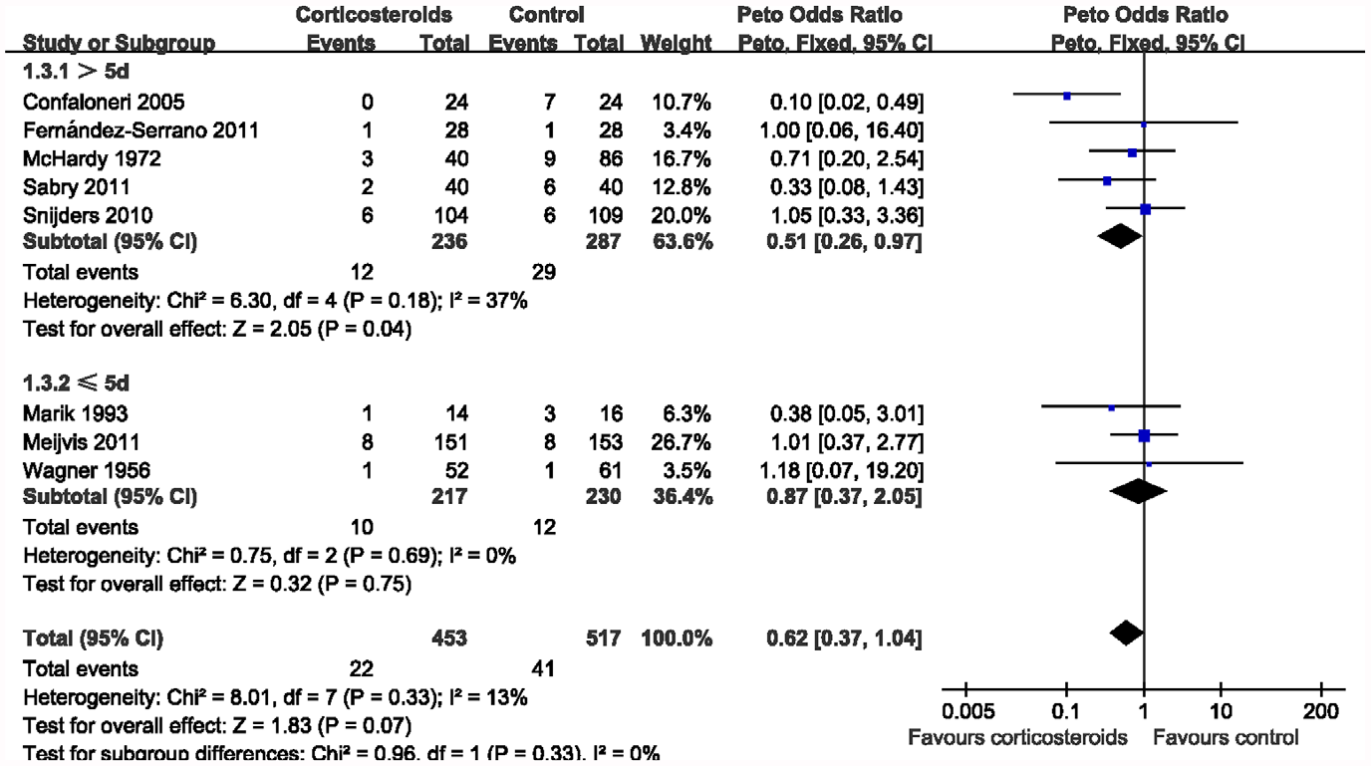
Subgroup analysis according to the duration of corticosteroids treatment.

In the subgroup analysis by the severity (**Figure 3**), significant association was found among severe CAP patients and mortality (Peto OR 0.26, 95% CI 0.11–0.64; *P = *0.003; *I* ^2^ = 0%), but was not found among mild to severe CAP patients (Peto OR 0.95, 95% CI 0.50–1.78; *P = *0.86; *I* ^2^ = 0%). Subgroup analysis was also performed by duration of corticosteroids treatment. As shown in **Figure 4**, significant reduced mortality was found among patients with prolonged treatment (Peto OR 0.51, 95% CI 0.26–0.97; *P = *0.04; *I* ^2^ = 37%). However, the subgroup analysis of trials with shorter course failed to support the beneficial effect of corticosteroids in CAP (Peto OR 0.87, 95% CI 0.37–2.05; *P = *0.75; *I* ^2^ = 0%).

### Sensitivity Analysis

The sensitivity analysis using the fixed-effects model yielded estimates similar to those of the Peto odds ratio for the risk of mortality (RR 0.63, 95% CI 0.38–1.04; *P = *0.07; *I* ^2^ = 0%). The sensitivity analysis using a random-effects model yielded estimates similar to those of the Peto odds ratio for the mortality risk (RR 0.73, 95% CI 0.43–1.23; *P = *0.23; *I* ^2^ = 0%).

### Publication Bias

**Figure 5:**
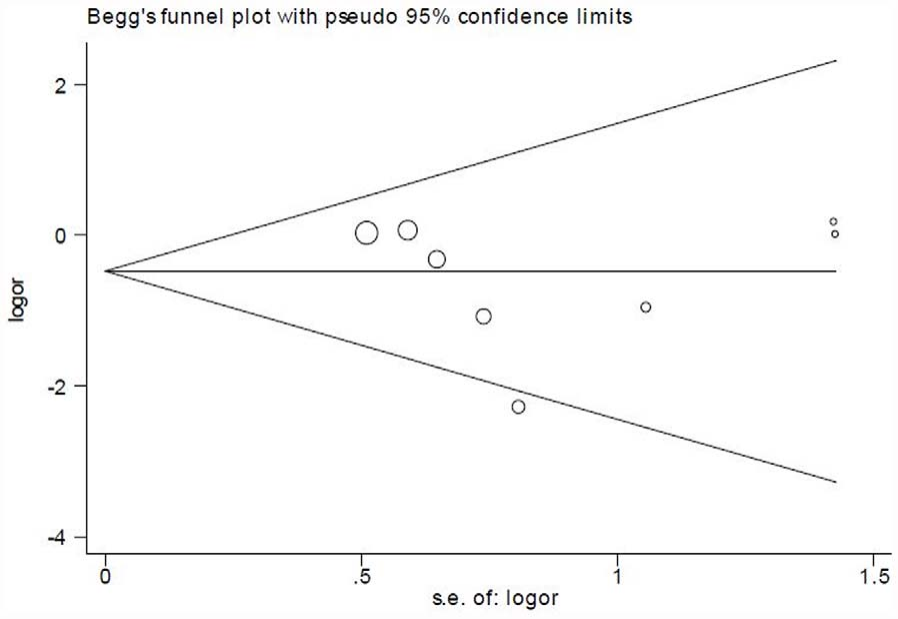
Funnel plot of the included trials for mortality.

The funnel plot for mortality showed slightly asymmetric (**Figure 5**). However, Egger’s test did not indicate significant publication bias (*P = *0.556).

## Discussion

In this current meta-analysis of 9 RCTs including 1001 patients hospitalized for CAP, mortality was not significantly reduced by the use of corticosteroids. In the severe CAP subgroup, however, we observed a statistically significant decrease in mortality associated with the use of corticosteroids. There could be two potential reasons for the observed survival benefit. First, the immunomodulatory and anti-inflammatory effects of corticosteroids may damp or attenuate the inflammatory response due to severe CAP. Corticosteroids are the most powerful inhibitors of inflammation. They can switch on genes that encode anti-inflammatory cytokines and switch off genes that encode pro-inflammatory cytokines [27], [28]. Second, in addition to the inflammatory response, the critical illness-related corticosteroid insufficiency (CIRCI) may play an important role in severe CAP. According to IDSA/ATS consensus guideline, CIRCI should be screened in all patients at risk for severe CAP [29]. In a systematic review, Salluh et al. [30] showed that the prevalence of CIRCI in severe CAP ranged from 0% to 48%. Cortisol is a major regulator in the immune system and inflammation. Therefore, corticosteroid replacement therapy might be effective in critical illness, including severe CAP. In subgroup analysis by the duration of corticosteroids treatment, we found that prolonged corticosteroids treatment (>5 days) was associated with a greater benefit compared with treatment courses less than 5 days. Recently, Annane et al. [23] assessed the use of corticosteroids for severe sepsis and septic shock in a systematic review. They showed that hydrocortisone for a prolonged duration (>5 days) may improve survival in severe sepsis and septic shock [23]. Moreover, a study on acute respiratory distress syndrome (ARDS) suggested that more than 7 days corticosteroids strategy led to reduction in markers of inflammation, duration of mechanical ventilation, and intensive care unit stay [31]. Therefore, the beneficial effect of prolonged corticosteroids treatment course on decreasing mortality in CAP cannot be excluded.

The potential side effects from corticosteroids in CAP should be clarified. Theoretically, corticosteroids could favor the onset of metabolic disorders, gastroduodenal bleeding, muscle weakness, and superinfection. Previous study found that corticosteroids increased the risk of hyperglycemia and hypernatremia [23]. In addition, there was no evidence for an increased risk of bleeding, superinfection, or neuromuscular weakness [23]. In our meta-analysis, treatment with corticosteroids in CAP was associated with an increased risk of hyperglycemia, but was not associated with gastroduodenal bleeding and superinfection. However, we could not address the association between treatment with corticosteroids and risk of hypernatremia or neuromuscular weakness. It was due to insufficient information can be extracted from primary publications. Further studies should be designed to analyze these issues. Hyperglycemia occurred frequently in corticosteroids treated patients. Berghe et al. [32] indicated that intensive blood glucose control reduced morbidity and mortality among critically ill patients. Therefore, the latest IDSA/ATS guideline suggested that close attention to tight glucose control was required, if CAP patients received corticosteroids [29]. A strict surveillance of control of blood glucose levels must be systematically conducted by physicians.

The results from meta-analyses could be influenced by publication bias. Egger’s test did not show significant publication bias. The asymmetric funnel plot, however, showed potential publication bias. Therefore, more studies are still needed to confirm the findings from this meta-analysis. Furthermore, there was no significant statistical heterogeneity in most of the comparisons. Thus, heterogeneity did not seem to have influenced the results. We also carried out a sensitivity analysis. The change of statistical models did not alter our conclusion of mortality, suggesting the reliability of this result.

Some limitations of this meta-analysis should be considered. First, the number of available studies that could be included in this meta-analysis was moderate. Therefore, the results could be influenced by the factors like random error. Second, this study was a study-level meta-analysis but not an individual patient-level meta-analysis. It is known that study-level analyses can lead to biased assessments and use of aggregated summary values has some limitations for explaining the heterogeneity [33], [34]. Third, because only studies that were indexed by the selected databases were included for data analysis, some relevant published studies or unpublished studies were missed, which may have biased our results.

In summary, although our overall results did not suggest a benefit for corticosteroids treatment in patients with CAP, analyses restricted to severe CAP patients or prolonged corticosteroids treatment showed a survival benefit. However, the number of studies only including severe CAP patients was small (4 studies, n = 214). Thus, large-scale, double-blind, placebo-controlled trials are still needed to evaluate the effects of corticosteroids in adults with severe CAP. A sensitive and validated formula to identify those who need corticosteroids in CAP patients is warranted. In addition, type of corticosteroids, the dosage, the duration, and tapering of the treatment, should be evaluated in rigorously designed and adequately powered RCTs in the future.
